# Developing an Integrative Treatment Program for Cancer-Related Fatigue Using Stakeholder Engagement – A Qualitative Study

**DOI:** 10.1177/1534735417740629

**Published:** 2017-11-21

**Authors:** Claudia Canella, Michael Mikolasek, Matthias Rostock, Jörg Beyer, Matthias Guckenberger, Josef Jenewein, Esther Linka, Claudia Six, Sarah Stoll, Roger Stupp, Claudia M. Witt

**Affiliations:** 1 University Hospital Zurich, Zurich, Switzerland; 2 University of Zurich, Zurich, Switzerland; 3 University Medical Center Hamburg-Eppendorf, Hamburg, Germany; 4 Zurich, Wilen bei Wollerau, Switzerland; 5 Cancer League Ostschweiz, St Gallen, Switzerland.; 6 Charité–Universitätsmedizin Berlin, Berlin, Germany; 7 University of Maryland School of Medicine, Baltimore, MD, USA

**Keywords:** cancer-related fatigue, stakeholder engagement, integrative treatment program, complementary medicine, qualitative study

## Abstract

**Background:** Although cancer-related fatigue (CRF) has gained increased attention in the past decade, it remains difficult to treat. An integrative approach combining conventional and complementary medicine interventions seems highly promising. Treatment programs are more likely to be effective if the needs and interests of the people involved are well represented. This can be achieved through stakeholder engagement. **Objectives:** The aim of the study was to develop an integrative CRF treatment program using stakeholder engagement and to compare it to an expert version. **Method:** In a qualitative study, a total of 22 stakeholders (4 oncologists, 1 radiation-oncologist, 1 psycho-oncologist, 5 nurses/nurse experts, 9 patients, 1 patient family member, 1 representative of a local Swiss Cancer League) were interviewed either face-to-face or in a focus group setting. For data analysis, qualitative content analysis was used. **Results:** With stakeholder engagement, the integrative CRF treatment program was adapted to usual care using a prioritizing approach and allowing more patient choice. Unlike the expert version, in which all intervention options were on the same level, the stakeholder engagement process resulted in a program with 3 different levels. The first level includes mandatory nonpharmacological interventions, the second includes nonpharmacological choice-based interventions, and the third includes pharmacological interventions for severe CRF. The resulting stakeholder based integrative CRF treatment program was implemented as clinical practice guideline at our clinic (Institute for Complementary and Integrative Medicine, University Hospital Zurich). **Conclusion:** Through the stakeholder engagement approach, we integrated the needs and preferences of people who are directly affected by CRF. This resulted in an integrative CRF treatment program with graded recommendations for interventions and therefore potentially greater sustainability in a usual care setting.

## Introduction

According to the World Cancer Report 2015, there will be 21.7 million expected new cancer cases and 13 million predicted cancer deaths by 2030.^1^ At the same time, there is a growing population of survivors as a result of more effective cancer treatments.^1^ Cancer-related fatigue (CRF) is one of the most common and distressing symptoms in cancer patients regardless of tumor and treatment type.^2,3^ Although there is no clear consensus regarding the definition of CRF,^4^ the National Comprehensive Cancer Network (NCCN) provides one of the most commonly used descriptions: CRF is “a distressing, persistent, subjective sense of physical, emotional and/or cognitive tiredness or exhaustion related to cancer or cancer treatment that is not proportional to recent activity and interferes with usual functioning.”^5^ Moreover, up to 95% of cancer patients experience CRF during chemotherapy or radiotherapy^6^ and face a high risk of experiencing CRF in the posttreatment phase^7^. CRF has been associated with shorter survival^8,9^ and a significant decrease in overall quality of life.^10^ Additional consequences are significant health care costs, staying off work or sick leave and lost earnings and productivity.^11-16^

At present, CRF is often undetected and untreated in many cancer patients.^4^ This might be because patients are often focusing on survival; consequently, they consider fatigue symptoms as an inescapable side effect of cancer therapy and therefore do not report them to their health care providers. Furthermore, lack of awareness and time pressure on health care providers may prevent them from detecting CRF symptoms.^17-19^ Moreover, limited energy and mobility are obstacles in making additional physician’s appointments that could unmask CRF symptoms.^20^

CRF is complex and has multiple causes. It is difficult to distinguish between the causative, intensifying and maintenance factors, which can manifest on different levels, such as on somatic, emotional, or cognitive level, in a given patient. Consequently, a careful differential diagnosis is needed when a cancer patient reports fatigue symptoms.^5,21,22^

After CRF is diagnosed, complex, non-pharmacological interventions are usually applied.^5^ In particular, psychosocial interventions and exercise can reduce CRF during cancer treatments.^5,23,24^ In addition, approximately 40% of cancer patients use complementary medicine (CM) interventions.^25^ In 2016, the NCCN provided an overview of the evidence regarding CM treatments for CRF, which the network integrated into its guidelines; the included CM treatments are yoga, bright white light therapy, and mindfulness-based stress reduction.^5^ Furthermore, the results of randomized controlled trials suggest that other CM interventions—namely, acupuncture, acupressure, ginseng, guarana, and qigong—may reduce CRF.^5,26^ However, because of its complexity, CRF remains difficult to treat, and a multimodal treatment program is required. An integrative approach that combines conventional and CM interventions, considers patients’ needs and preferences and provides opportunities for self-care seems highly promising. In addition, effects, possible side effects and interactions can be better monitored with an integrative approach.

Treatment programs are more likely to be effective if the needs, values, and interests of the people involved are considered.^27-29^ This can be achieved with stakeholder engagement. In the medical field, stakeholder engagement means engaging, for example, patients and their caregivers, patients’ family members, and health care providers but also other stakeholders, such as patient advocacy and support groups, research funding agencies, cancer leagues, and other funders of medical care. Surroundings, such as infrastructures, and context factors (eg, political environments) are also considered. In short, everyone affected by and involved with a specific topic can provide input.^27-29^

Including stakeholder engagement in the research process increases the chance that the topic of a study addresses the questions and needs of the stakeholders and that the results will contribute to improved practices.^26,28,30,31^ Patients suffering from CRF are dealing with complex interactions among the symptoms, effects and side effects of the disease and its different treatments. The subjective experience of these complex relationships can only be adequately reported by the patients themselves and by those who are directly affected by it.^30^ Therefore, developing a coherent treatment program that combines CM interventions with conventional therapies for CRF requires the knowledge and integration of the stakeholder’s experiences, needs, and values.

The aim of this study was to develop an integrative CRF treatment program using stakeholder engagement and comparing it with an expert version of the program.

## Methods

**Figure 1.**
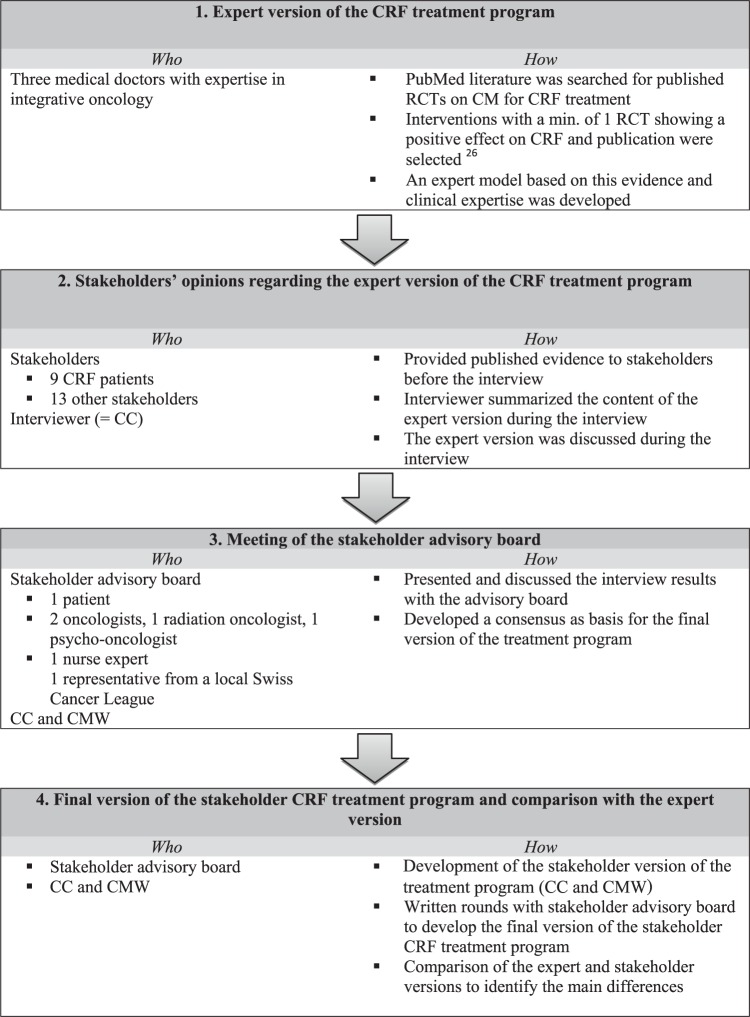
From the expert version to the stakeholder version of the cancer-related fatigue (CRF) treatment program.

We used a stepwise approach to develop the final stakeholder version of the CRF treatment program (Figure 1).

We started with the development of a literature- and evidence-based expert version of an integrative CRF treatment program. To develop the stakeholder version of the treatment program, we conducted qualitative face-to-face and focus group interviews with the stakeholders. The results from the interviews were presented to the stakeholder advisory board, and a consensus was developed. The stakeholder treatment program was finalized in written Delphi rounds and compared with the expert version.

The project was approved by the ethics committee of Zurich (KEK-ZH-Nr. 2014-0689), Switzerland, in March 2015 and was conducted accordingly. All the participants provided written informed consent.

### Stakeholder Engagement

We involved different stakeholders (oncologists, a radiation oncologist, a psycho-oncologist, nurses, nurse experts, a representative of a local Swiss Cancer League [an expert consultant in cancer survivorship], patients, and a patient’s family member) to learn about their experiences and needs concerning CRF and to gather their opinions and suggestions on the expert version of the integrative CRF treatment program.

In addition, we formed a stakeholder advisory board consisting of 1 patient, 2 oncologists, 1 radiation oncologist, 1 psycho-oncologist, 1 nurse expert, and 1 representative from one of the local Swiss Cancer Leagues (an expert consultant in cancer survivorship). The members of the advisory board not only represented the different stakeholder groups involved in the project but were also very experienced with CRF.

The stakeholders were mainly recruited from within the University Hospital Zurich according to the principles of “theoretical sampling.”^32^ The health care providers represented the hospital’s main oncology departments, whereas the patients came mostly from our clinic (Institute for Complementary and Integrative Medicine, University Hospital Zurich). To complete the sample and ensure that we obtained different experiences with and perspectives on CRF, some stakeholders (eg, the patient family member, the representative from a local Swiss Cancer League and a nurse expert specialized in CRF) were recruited from outside the clinic.

### Qualitative Data Collection and Analysis

Data collection and analysis followed the principles and methods of qualitative content analysis.^33-35^ We used qualitative content analysis to focus on the whole spectrum of topics and viewpoints (eg, in contrast to discourse analysis, which focuses more the ongoing of a discussion) that the interviewees brought up regarding the experiences and needs with CRF. As a first step, the stakeholders were interviewed either face-to-face or in a focus group setting.^33-35^ The qualitative interviews were semistructured and lasted approximately 90 minutes. They consisted of 2 main parts. In the first part, the stakeholders were asked open questions about their experiences and needs concerning CRF treatment (patients) or about their opinions regarding the treatment options for CRF (other stakeholders) of which they are aware. In the second part, the stakeholders were asked to evaluate the expert version of the integrative CRF treatment program. All the interviews were audio recorded.

As the second step, 2 researchers from our clinic (Institute for Complementary and Integrative Medicine, University Hospital Zurich) independently summarized the main topics that the stakeholders brought up by listening to the audiotaped interviews. They validated their results intersubjectively in a discussion and merged them in a separate summary. In addition, based on their validation, the transcription and coding order was determined. This process corresponds to the “video club” approach, which has its origin in education research.^36^ In this approach, teachers discuss their videotaped lessons in groups to improve their teaching. The basic assumption behind a video club is that the members gain a more profound and informed understanding of the data by dealing with real-time data and discussing their perceptions and interpretations with each other.

In the next step, the audio-recorded interviews were transcribed.^37,38^ The same 2 researchers inductively built a thematic code system for the data according to the principles of grounded theory’s “constant comparison method.”^32,39^ The data were then coded in sense units using the qualitative data analysis software MAXQDA, Version 11.1.2. The coding was interchangeably validated by the 2 researchers and then analyzed in relation to the further development of the expert version of the integrative CRF treatment program.

## Results

### Sample

In total, 22 stakeholders (13 women, 9 men) were interviewed, resulting in approximately 21 hours of audio data. The involved stakeholders were 9 patients and 13 other (4 oncologists, 1 radiation oncologist, 1 psycho-oncologist, 5 nurses/nurse experts, 1 representative of a local Swiss Cancer League and 1 patient’s family member).

With the patients (9 in total, 7 women, 2 men; mean age of 55 years, range 35-65 years), we conducted 2 face-to-face interviews and 1 focus group consisting of 7 participants (Table 1).

**Table 1.** Sample Patients: Cancer Characteristics.

Cancer Characteristics	Specification	Number
Diagnosis	Anal	1
	Breast	3
	Colon	1
	Gastrointestinal stromal tumor	1
	Prostate cancer	1
	Lymphoma	2
	Aggressive non-Hodgkin’s lymphoma	(1)
	Hodgkin disease	(1)
Years since diagnosis	≥9	1
	3-4	3
	1-2	4
	≤ 1	1
Treatment	Surgery	6
	Chemotherapy	7
	Radiation therapy	5
	Hormonal therapy	2
	Immunotherapy	1
	Targeted therapy	1
Metastasis	Yes (liver)	1
	No	8
Tumor recurrence	Yes	1
	No	8
Previous cancer	Yes, Hodgkin disease (1986), osteoma (2006)	2
	No	7

The average time span between the patients’ cancer diagnoses and their CRF diagnoses was 10 months. During the project, we confirmed the CRF diagnosis of the participating patients using a numeric rating scale with 2 questions.^22^ First, the patients rated the intensity of fatigue within the past week on a scale from 0, no fatigue, to 10, the most severe fatigue imaginable (question 1). Second, the patients were asked to rate how their fatigue affected their daily life during the past week using a similar 0-to-10 scale (question 2). Generally, if the intensity was rated 4 or higher for the first question and 5 or higher for the second, further diagnostics should be considered. The patients showed an average score of 5.1 (range 2-8) for question 1 and 4.6 (range 0-9) for question 2. All the patients had either completed or were undergoing standard cancer therapy and had previously used CM treatments.

With the other stakeholders (6 women, 7 men; mean age of 45 years, range 31-62 years), we conducted 10 face-to-face interviews and 1 focus group consisting of 3 nurses and nurse experts. The interviewed health care providers estimated that approximately 57% (range 10%-100%) of their cancer patients suffer from CRF.

The stakeholder advisory board included a subset of the 22 stakeholders. With the exception of the interviewer and the principal investigator (CC and CMW), the individual board members were interviewed face-to-face as stakeholders, independent of the advisory board meeting.

### Expert Version of the Integrative CRF Treatment Program

The expert version of the integrative CRF treatment program ^26^ was derived from the available scientific evidence.^26^ It introduces individual interventions as treatment options on the same level and presents the possibility of combining 2 or more of them if needed (Table 2).

**Table 2.** Expert Version of the Integrative Cancer-Related Fatigue (CRF) Treatment Program Before Stakeholder Engagement.

Process	Intervention	Focus
Use one or more treatments	Psychoeducation	Activity and rest, sleep, opportunities, and limits
	Exercise	Endurance, strength, fun
	Mind body medicine techniques	Mindfulness meditation, autogenic training, progressive muscle relaxation, yoga, qigong
	Acupressure and acupuncture	Acupressure: daily as self-care Acupuncture: 1×week, 6 weeks
	Medication	Methylphenidate, modafinil, corticosteroid, ginseng, guarana

During psychoeducational intervention, patients are informed about CRF, and then specific topics such as activity and rest, sleep in general, possible comorbidities such as depression or anxiety, and new opportunities and limits under CRF are discussed.^40^ This intervention is usually provided by psycho-oncologists.

Patients are encouraged to participate in the whole treatment program, which consists of different interventions.^41^ One intervention encourages patients to improve endurance and strength by choosing a form of exercise that they enjoy.^42,43^ Another intervention consists of mind body medicine techniques, which include classic relaxation techniques such as autogenic training or progressive muscle relaxation, and mindfulness-based approaches such as mindfulness meditation, yoga, or qigong.^44-47^ Patients may also benefit from a total of 6 weekly individual acupuncture treatments based on traditional Chinese medicine.^48,49^ Moreover, patients are theoretically and practically introduced to acupressure, including 5 points that have a relaxing effect: heart 7, liver 3, spleen 6, anmian, and yintang.^50^ The points should be stimulated daily for 3 minutes each (the first 4 points on both sides of the body and yintang as a single point). The patients also receive a handout describing acupressure concepts so that they can practice at home after the encounter with the specialist. There are also several drugs available to treat CRF, including methylphenidate, modafenil, corticosteroids, ginseng, and guarana.^51-56^ Patients are introduced to their modes of action, side effects, and effectiveness and explore what types of medication could be useful for their specific condition.

### General Needs Regarding an Integrative CRF Treatment Program

The stakeholders (all the terms in quotation marks indicate original quotes from the interviews with the stakeholders) indicated a need for patient orientation and agreed that strengthening “autonomy” and “self-determination” was an overall goal of CRF treatment. In their opinion, patient orientation in CRF treatment would also involve creating a “drop-in center” that “coordinates” and “monitors” and “where there is time to answer patients’ questions” and that is “geographically a comfortable distance” from patients. In this context, the stakeholders also called for stronger interdisciplinary cooperation among all involved health care providers. Moreover, the patients expressed their wish to be “closely supported” and “accompanied” both during therapy and after active treatment ends. The patients reported having suffered from substantial fatigue that restricted their activities of daily living and work and said that they often felt “left alone”, particularly after the active cancer treatment period had ended. Consequently, they would have liked “more frequent aftercare”. The nurses suggested that in such an environment, they could provide a “hinge-like function” if they were adequately empowered. Many stakeholders would welcome “applying a standard diagnostic tool for CRF” rather than being presented with numerous different tools that lack uniformity and validation.

### Overall Opinion Regarding the Expert Version of the Integrative CRF Treatment Program

In general, the interviewed stakeholders thought that CRF should be addressed preventatively immediately after the cancer diagnosis. The stakeholder advisory board further discussed what information should be given at what time point and concluded that patients should be informed soon after diagnosis but not during the initial consultation immediately after diagnosis because at that appointment, patients usually are overwhelmed and have a hard time processing all the information they receive. The advisory board would prefer the creation of an online information tool about CRF for the cancer patients.

According to the stakeholders, self-care options should be at the center of the interventions, motivating patients to be active and to regain self-efficacy. The patient family member summarized this need as follows: “…something that you can do by yourself …nothing that is additionally inflicted upon you”. Patients and the patient family member emphasized the importance of being active themselves but also noted they often lack important information about CRF. The nurses in particular, but also other stakeholders, stressed that the treatment program should be individualized and should consider the personal resources, interests and cultural and social background of the patients before their cancer diagnosis. In the stakeholders’ opinion, the treatment program will be better integrated into patients’ everyday lives if it fully considers the patient as an individual.

The stakeholder advisory board suggested the widespread use of the CRF treatment program but assumed that the program would be more attractive to patients who are “open to CM”, such as those who are “young”, more “active” and have an “awareness of self-care”. For the “average cancer patient” who is “older than 65”, “inactive, with “no awareness of self-care” and suffering from “comorbidities”, the CRF treatment program would require a “complete change of lifestyle” that would be very difficult to achieve in the context of CRF. Consequently, the board members discussed whether a target group should be defined for the proposed treatment program. As they wished for the program to experience widespread use, they suggested screening all cancer patients for CRF and then following in a patient-oriented manner rather than defining a target group.

### Introducing Levels and Prioritizing Treatments

The health care providers and the patient family member called for “prioritizing the different treatment options in the program” to provide clearer guidance and to take into account the exhaustion and tiredness of the CRF patients, which could make fewer interventions be more feasible. In comparison, the patients pointed to the importance of patient orientation, “adjusting the treatment to the individual” and “his or her everyday situation”. The nurses added the importance of also considering the patient’s “social and cultural background”. Briefly, the stakeholders stressed prioritizing approaches and patient orientation.

The stakeholders agreed that psychoeducation and exercise should be the first priority; these approaches were considered the most effective for treating CRF, and the 2 treatments were deemed equally valuable. Furthermore, all stakeholders preferred non-pharmacological treatment options to pharmacological treatment options. They found mind body medicine techniques reasonable overall but had conflicting opinions regarding acupuncture. Whereas some interviewees were critical of acupuncture’s effectiveness, others reported having positive experiences with acupuncture.

### Priorities When Starting the CRF Treatment Program

In terms of priorities, the stakeholders agreed on 2 interventions with which the treatment program should start. First, patients and their family members should be informed about CRF soon after their cancer is diagnosed. At present, cancer patients are not usually systematically informed about CRF. Several patients commented that they had to “find out everything by themselves”, which was difficult while suffering from CRF. Additionally, the stakeholders stated that being informed about the existence of CRF and “what it means to suffer from CRF” helps patients better “understand”, “accept” and “cope” with the syndrome. In addition, “autonomy” and “self-determination” can only be achieved “with good-quality information”, according to the stakeholders.

Second, patients should start exercising soon after they are diagnosed with cancer. A nurse specified that “cancer patients in a curative and palliative condition should be differentiated.” Based on her experience, CRF patients receiving curative treatment should be informed about CRF and start exercising as described above, whereas CRF patients receiving palliative care should “focus on coping”, for instance, by “setting priorities”, “managing their energy”, or “learning to let go.”

### Stakeholders’ Thoughts About Individual Treatment Options

#### Psychoeducation and Exercise

Although the stakeholders stated that psychoeducation was 1 of the 2 most important first steps for treating CRF, they found the term itself problematic:

Nurse 1: At the very moment they [the patients] hear “psycho”, doors are closing immediately and we don’t need to discuss it any further…”

Nurse 2: …Could we use the popular term “empowerment” or “promoting self-management?” You know, to cancel the “psycho.” Because, I mean, we are health specialists. I am thinking about it as information. However, if I’m showing it [the treatment program] to a patient and “psychoeducation” is written on it—and least of all, I want to be educated as a patient.

Therefore, the stakeholders opted to change the term “psychoeducation” to avoid the stigma of suffering from a “mental disorder” and the implicit moral statement that the patient somehow needs to be educated.

Along with psychoeducation, the stakeholders considered exercise a first priority when treating CRF. The patient family member added that caregivers and family members can exercise with the patient and that this provides an opportunity to contribute actively to the patient’s health. This was very important to the interviewed patient family member as a means of overcoming the “helplessness” that the partner of a cancer patient could feel.

Stakeholders associated regaining trust in the body and preserving muscle mass as important goals:

Medical doctor: What you shouldn’t underestimate in exercise and strength is gaining back trust in one’s own capacity. If they couldn’t take three steps on the stairs and suddenly, after one month, they manage the stairs.

Furthermore, regaining physical capacity was thematically linked to regaining self-efficacy:

Nurse expert: People are telling me that they cannot trust their own body because they suffer from cancer and didn’t notice it. Then, fatigue comes… and surgery, being disfigured, not being able to consider yourself beautiful anymore. So, distance from your own body. Sometimes, they look at themselves from the outside and say, “That is someone different. I do not want anything to do with that”…And yes, afterwards, you should return to life, but it does not work out…It’s very important to give people back these capacities, that they can influence their own actions and experiences.

This nurse expert summarized what most of the stakeholders reported: that patients lose their faith in their body and how important it is for them to regain self-efficacy. The interviewees believed that patients could rebuild trust in their body and regain self-efficacy through a combination of psychoeducation and exercise.

Regarding exercise, the stakeholders also highlighted the problem that starting to exercise while suffering from fatigue is difficult. The health care providers thought that exercising with CRF is easier for patients were physically active before their cancer diagnosis. They considered the program proposed in the expert version more realistic for patients with light to moderate CRF. The patients pointed to the difficulty of exercising during or soon after chemotherapy because of its side effects and the resulting exhaustion. Some of the patients said they liked to be coached or at least guided during exercise:

Patient: The doctors suggest [concerning exercise] to do whatever feels good to you …That is something for the fifty-plus…I would have needed coaching.

The health care providers suggested institutionalized exercise programs. The advisory board members specifically preferred institutionalized group programs to individual coaching because of better cost-effectiveness and the limited resources of the health care providers.

#### Mind Body Medicine Techniques

The stakeholders welcomed mind body medicine techniques in treating CRF. Some of the health care providers went into more detail and described mind body medicine techniques as a “way to process cancer” and all its side effects, including CRF; they suggested that these techniques could help patients “cope with the stress”, “the emotions”, and “the existential questions” that come along with suffering from cancer. A nurse described this effect as follows and added that mind body medicine techniques might carry less stigma than psychotherapy:

Nurse: …it is a way for these people [cancer patients] to retreat to the quiet and not try to suppress anything; it is a way to open oneself to emotions . . . It becomes apath for processing…It carries less stigma than going to a psychologist.

Whereas some stakeholders believed that mind body medicine techniques only work when patients had already practiced them before their cancer diagnosis, other stakeholders reported that they had benefited from mind body techniques, although not all of them had practiced them before their cancer diagnosis.

#### Acupuncture and Acupressure

The stakeholders had conflicting opinions regarding acupuncture and acupressure. Some of the medical doctors and nurses were rather critical, stating that acupuncture and acupressure are more a “question of belief” than an effective therapy. A medical doctor explained his rejection of acupuncture in the context of his worldview:

Medical doctor: That you puncture some energy lines or whatever and points—it doesn’t fit at all into my philosophical and physiological worldview. It simply doesn’t fit in my concept of body functions and physiology.

In addition, these stakeholders were often not convinced that the existing clinical trials are of good quality.

In contrast, there were stakeholders who reported experiencing positive effects of acupuncture and acupressure on CRF, such as the following patient:

Patient: Acupuncture is—during an episode of fatigue, if you are really inside [the fatigue], not just at its border area, if you really suffer from fatigue, then acupuncture is awesome!…It gives me energy. I react very well to it.

Concerning acupressure, the stakeholders welcomed the possibility of a treatment that “can be done by the patients themselves at home,” allowing them to actively “contribute to their own well-being” and “strengthen their autonomy”. Two patients mentioned that practicing acupressure is not always easy. One patient reported that she “immediately falls asleep” while performing acupressure on herself and that she was therefore unable to execute it. The other patient mentioned a “lack of strength in her arms” as a result of chemotherapy that would have left her unable to perform acupressure.

#### Medication

The stakeholders agreed that medication should be the last level of the treatment program. They argued that patients “refuse to swallow more drugs than they already have to take.” A patient described the following feeling: “I could not bear to even see another pill [during chemotherapy], not to mention swallowing one.” Although the medical doctors and nurses noted that steroids and psychoactive drugs had short-term effects on CRF in their patients, they also observed severe side effects. A medical doctor described these observations as follows: “Corticosteroids may have a good albeit short-lived effect in some patients. However, you are buying in a side effect through the back door.” The interviewed doctors and nurses warned of possible interactions between the different drugs that are usually used during cancer therapy. In addition, they were critical of the existing clinical trials on the effects of all the proposed medications on CRF.

### Integrative CRF Treatment Program After Stakeholder Engagement

Based on the presented stakeholder evaluation and its discussion among the stakeholder advisory board, the integrative CRF treatment program was further developed. The most important issue seemed to be the adjustment of the program to the stakeholders’ wish to prioritize treatment options and include patient preferences and resources in treatment selection (see Table 3).

**Table 3.** Cancer-Related Fatigue (CRF) Treatment Program After Stakeholder Engagement.

Level	Process	Intervention	Focus
1	Main treatment for all patients, including advice for implementation	Information and motivation	Activity and rest, sleep, opportunities, and limits
		Exercise	Endurance, strength, fun
2	Introduced as additional treatment options and used according to patient’s choice	Mind body medicine techniques	Mindfulness meditation, autogenic training, progressive muscle relaxation, yoga, qigong
		Acupressure and acupuncture	Acupressure: daily as self-care Acupuncture: 1×/week, 6 weeks
3	Offered only to patients with severe CRF	Medication	Methylphenidate, modafinil, corticosteroid, ginseng, guarana

The stakeholders’ needs for prioritization was reflected in the new treatment program by clearly structuring the individual interventions into single steps in descending order of priority. The first level comprises providing information to patients and caregivers about CRF and the potential benefit of exercise. Access to this basic information should be mandatory for all patients. Patients should be informed about the second level of options, which includes mind body medicine techniques, acupressure and acupuncture that can be used according to patient preference. Medication (the third level) should be only used in cases of severe CRF.

To ensure awareness and widespread use of the program, all cancer patients should be at least screened for and informed about CRF.

Because of the stakeholders’ concerns about the possible stigma associated with the term “psychoeducation”, that intervention was renamed “information and motivation”.

#### From the Expert Version to the Stakeholder Version of the Integrative CRF Treatment Program

**Figure 2.**
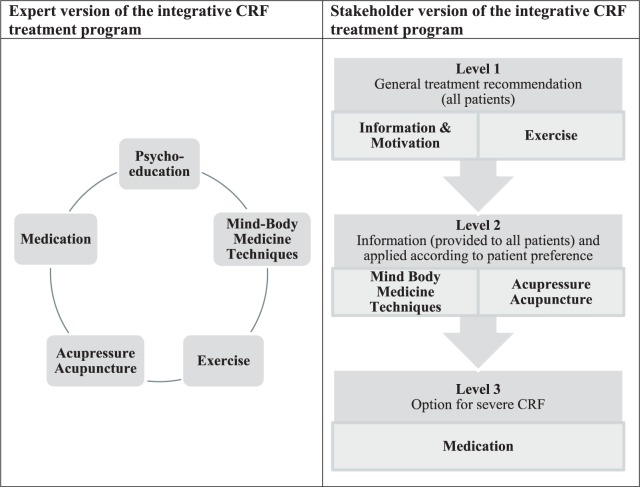
The expert and stakeholder versions of the integrative cancer-related fatigue (CRF) treatment program.

In summary, starting with the expert version of the integrative CRF treatment program, which placed a number of treatment options on the same level, the new stakeholder version clearly prioritizes the interventions by introducing 3 different treatment levels, with level 2 taking patient preferences and resources into account (Figure 2).

## Discussion

Overall, there was broad agreement among the interviewed stakeholders regarding their experiences and needs in relation to CRF and their opinion on the expert version of the integrative CRF treatment program. This agreement facilitated the further development of the treatment program and increased the probability that the program will be feasible and cover the needs of different stakeholder groups. Compared with the expert program, the stakeholder engagement process first and foremost resulted in an emphasis on patient orientation on different levels: adjusting to the real-life situation by prioritizing the individual treatment options and considering the resources and interests of the individual patients by offering a variety of treatment options at the second level of priority.

The resulting stakeholder based integrative CRF treatment program was implemented as clinical practice guideline at our clinic (Institute for Complementary and Integrative Medicine, University Hospital Zurich). The first experiences indicate that the program is very feasible in informing the patients about the treatment options as well as starting with a manageable treatment for the respective CRF patient.

Our approach had advantages and limitations. The advantages were the inclusion of a stakeholder advisory board that included relevant stakeholder groups, the use of a stepwise systematic approach to develop the treatment model and the application of robust qualitative research methods. We conducted the stakeholder engagement mainly in the context of our hospital, which offered the advantage of developing a consistent model and a feasible program but had the limitation of possibly being too tailored and not applicable to other settings or health care systems. However, because a multimodal treatment program is best and is usually offered by a multiprofessional team, the setting of a hospital with outpatient services seems adequate. We did not interview health insurance workers and hospital administrators, who might have provided other insights. The participating stakeholders based their statements on their own personal experiences and opinions, and a different group of stakeholders might have had different experiences.

We found the stakeholder engagement very helpful. In general, stakeholder engagement can help the people involved with CRF care make more informed health care decisions by identifying CRF as a critical and relevant research topic and by providing evidence-based information about treatment options, which are discussed with the patient and adapted to their individual situation, needs and resources. This corresponds with the general goals and advantages of stakeholder engagement supported by the Patient-Centered Outcomes Research Institute (PCORI), which was authorized by the US Congress in 2010 and had a budget of approximately $400 million in 2016.^27-29^

We concluded that the stakeholders’ emphasis on prioritizing information, exercise, and mind body medicine techniques corresponds with the current NCCN Clinical Practice Guidelines in Oncology for CRF.^5^ Whereas the guidelines also include bright white light therapy,^5^ our suggested treatment program adds more CM interventions to the NCCN Guidelines; namely, qigong, acupressure, acupuncture, ginseng, and guarana. This inclusion corresponds to the needs and practice of approximately 40% of cancer patients, who wish to include CM in their cancer treatment.^57^

### Challenges in the Implementation of the CRF Treatment Program

A major challenge in patient orientation is how the individual CRF patient can receive understandable, high-quality evidence-based information at the right moment. Stakeholders call for patients to be informed about CRF and treatment options soon after their diagnosis. The patients in the stakeholder group thought this information would be best provided by a drop-in center at the clinic that monitors and coordinates the treatment. The stakeholder advisory board discussed the issue of the right time point for introducing information about CRF and suggested creating an online tool that patients can access after they learn about CRF from the health care provider. Everyone seemed to agree that information about CRF should be provided soon after a cancer diagnosis. The advisory board further noted the problem that in some places, cancer care is based in different departments, which makes it difficult to create a drop-in center for patients. The right time point for discussing CRF remains an open question that has also not been answered by the NCCN Clinical Practice Guidelines in Oncology for CRF.^5^ Considering that CRF is complex and highly dependent on the patient’s individual cancer progression, the NCCN Guideline for CRF suggest that every clinic or institution should work out a procedure for how to best inform patients depending on the institution’s structures and resources.^5^ Oncology nurses, who evaluated a former draft of the guidelines, noted that patients often do not report their symptoms to their health care providers because they do not want to complain in general and they fear that fatigue is a sign of progressive cancer.^58^ Based on this, the authors recommend addressing CRF in a discussion between patients and health care providers.^58^

Another major challenge is the apparent contradiction and incompatibility between suffering from fatigue and getting active. It is hard for patients with CRF to overcome their exhaustion and find the motivation to exercise. The stakeholders suggested that this is best achieved when the CRF patients are guided or coached. This view is supported by current research, which found that supervised exercise has greater beneficial effects on CRF than exercise without supervision.^59,60^ Furthermore, the implementation of a supervised exercise program depends greatly on existing infrastructures and resources. Therefore, the stakeholder advisory board considered group programs or referrals to exercise specialists (eg, physical therapists) to be the most realistic intervention, but there is a lack of corresponding research to support or disprove the stakeholders’ opinion.^5,43,61^ Thus, future research is needed to determine how best to develop an institutionalized individual or group exercise program for CRF patients that considers all the relevant contextual factors, such as infrastructures, costs and resources.

A basic condition for mastering all the challenges in implementing such a treatment program is the need for education and training programs in CRF management for health care providers in oncology. Furthermore, CRF treatment should be reimbursed by medical care contracts.^5^

### Implications for Research and Practice

Further research should use quantitative methods to evaluate the acceptance of this treatment model by a wider audience. As a next step in our clinic, the implemented integrative CRF treatment program should be scientifically evaluated. Ideally, further qualitative research that considers not only the improvement in CRF and quality of life but also addresses questions about context factors, such as cost-effectiveness, infrastructure, and resources, should also be conducted.

## Conclusion

Although CRF has gained increased attention in cancer research and treatment in the past decade, it remains prevalent and difficult to treat. The presented integrative treatment program uses a multimodal approach that is structured into levels and combines effective conventional and CM treatments for CRF. By adopting a stakeholder engagement approach, we integrated the values, needs and preferences of people who are directly affected by or involved with CRF in the development of the treatment program to improve the quality, relevance, and feasibility of CRF treatment. Providing evidence-based information as an integral part of the treatment program will help the people affected by CRF to make informed health care decisions and potentially improve the health of the CRF patients. A qualitative research method approach was combined with stakeholder engagement to provide deep and rich insights into stakeholders’ individual circumstances, needs, and values.
